# Supplementary material for: Two surveys separated by almost a decade reveal the inexorable decline in sleep time in teens: Results of the National survey of middle and high schools in adolescents on health and substances (EnCLASS 2018), and the evolution since 2010
Source: PLoS One. 2025 Mar 31;20(3):e0314815. doi: 10.1371/journal.pone.0314815 (PMC11957357; doi:10.1371/journal.pone.0314815)
Supplement: S1 File — (PDF) [file pone.0314815.s001.pdf]

Human Participants Research Checklist

Leger D. Inexorable decline of sleep in teens 13-06-24

Did you obtain ethics approval for this study?

- If yes, please upload (file type “Other”) the original approval document you received from your ethics committee. If the original document is in another language, please also provide an English translation.

  x_ Uploaded ___ N/A

- If you did not obtain ethical approval, please explain why this was not required below.

Epidemiological surveys do not need an approval from an ethical committee in our country, but an approval from the CNIL (National commission on informatics and liberty. Document we joined in French and translated.

2. If you prospectively recruited human participants for the study – for example, you conducted a clinical trial, distributed questionnaires, or obtained tissues, data or samples for the purposes of this study, please report in the Methods:

   i. the day, month and year of the **start and end** of the recruitment period for this study.

      - For the first study from 01-11-2010 to 31-07-2011
      - For the second study: from 01-2017 to 31-07-2018

   ii. whether participants provided informed consent, and if so, what type was obtained (for instance, written or verbal, and if verbal, how it was documented and witnessed). If your study included minors, state whether you obtained consent from parents or guardians. If the need for consent was waived by the ethics committee, please include this information.

   _x_ Completed ___ N/A

   According to the hbsc study protocol document joint, parents/gardians and children have to be fully informed on the survey and were invited to sign if they did not want their child participate. They had access to the full questionnaire. See the French and translated versions of the documents.

3. If you are reporting a retrospective study of medical records or archived samples, please report in the Methods section:

   i. the day, month and year when the data were accessed for research purposes

   ii. whether authors had access to information that could identify individual participants during or after data collection

   ___ Completed ___ N/A

hbsc

HEALTH BEHAVIOUR IN SCHOOL-AGED CHILDREN (HBSC) STUDY PROTOCOL:

BACKGROUND, METHODOLOGY AND MANDATORY ITEMS FOR THE 2013/14 SURVEY

5. Ethical practice

WHO states that "all research involving human participants must be conducted in an ethical manner that respects the dignity, safety and rights of research participants and that recognises the responsibilities of researchers".[23] Children's rights are also specifically protected through the United Nations Convention on the Rights of the Child.[24] Article 12 addresses children's rights to express their views on all matters that affect them: it is expected that efforts be made to obtain informed consent from children involved in research projects, as well as their parents or guardians. HBSC recognises and adheres to these recommendations at each stage of the survey process.

Each HBSC country is required to:

1. ensure procedures are in place to review ethical conduct, often through an ethics committee within a university or region; where ethics committees are not in place, countries should adhere to national ethical guidelines concerning research with children and submit their protocol to any relevant board at country level;
2. make certain that any applicable legal requirements in relation to researchers working with children (such as police checks and police clearance certificates) are satisfied;
3. guarantee that study participants and their schools, parents/guardians are fully informed about the research and procedures are in place to enable them to withdraw from the study easily;
4. employ written and/or oral procedures for "informed" consent; and
5. fully document their national procedures.

Documentation is provided to inform parents/children of the ways in which confidentiality and anonymity are assured, giving details of who has access to the data and how they are stored and used. Explanations are provided in a way that children can understand. Parental (or guardian) and pupil consent is sought, as the young people involved are normally under the age of legal consent.[25] Informed consent relies on the quality of the information given and procedures in place to ensure the process is monitored. The approach typically adopted in HBSC is of "opt-out" or "passive" consent, with the option to withdraw from participation.

Instructions for those administering the survey highlight the importance of ensuring children are aware that they can choose whether or not to participate. Children are informed at the beginning of the survey that they do not have to answer questions if they do not want to.

Schools may wish to see the full questionnaire and it is useful if this is accompanied by a rationale for the study as a whole, a timescale, a description of what the study will entail in terms of time and teacher/pupil involvement and contact details of the research team. If schools are unhappy and want to exclude certain questions, HBSC teams respect this decision but record it for data-coding purposes.

CNIL.
3 Place de Fontenoy - 75334 PARIS Cedex 07
T. 01 53 73 22 22 - F. 01 53 73 22 00
www.cnil.fr

Monsieur MOREL DARLEUX Julien
OBSERVATOIRE FRANÇAIS DES DROGUES ET DES TOXICOMANIES
3 AVENUE DU STADE DE FRANCE
93200 LA PLAINE SAINT DENIS

RÉCÉPISSÉ

DÉCLARATION NORMALE

Numéro de déclaration

2155714 v 0

du 21 février 2018

A LIRE IMPERATIVEMENT

La délivrance de ce récépissé atteste que vous avez transmis à la CNIL un dossier de déclaration formellement complet. Vous pouvez désormais mettre en oeuvre votre traitement de données à caractère personnel.

La CNIL peut à tout moment vérifier, par courrier, par la voie d'un contrôle sur place ou en ligne, que ce traitement respecte l'ensemble des dispositions de la loi du 6 janvier 1978 modifiée en 2004. Afin d'être conforme à la loi, vous êtes tenu de respecter tout au long de votre traitement les obligations prévues et notamment :

1) La définition et le respect de la finalité du traitement,
2) La pertinence des données traitées,
3) La conservation pendant une durée limitée des données,
4) La sécurité et la confidentialité des données,
5) Le respect des droits des intéressés : information sur leur droit d'accès, de rectification et d'opposition.

Pour plus de détails sur les obligations prévues par la loi « informatique et libertés », consultez le site internet de la CNIL : www.cnil.fr

Organisme déclarant

Nom : OBSERVATOIRE FRANÇAIS DES DROGUES ET DES TOXICOMANIES

Service : PÔLE ENQUÊTES ET ANALYSES STATISTIQUES

Adresse : 3 AVENUE DU STADE DE FRANCE

Code postal : 93200
Ville : LA PLAINE SAINT DENIS

N° SIREN ou SIRET :
180036105 00039

Code NAF ou APE :
8411Z

Tél. : 0141627716
Fax. : 0141627700

Traitement déclaré

Finalité : ENQUÊTE SUR LES COMPORTEMENTS ADOLESCENTS

Fait à Paris, le 21 février 2018
Par délégation de la commission

Isabelle FALQUE PIERROTIN
Présidente

CNIL Authorization

2155714vo

DRUG AND DRUG ABUSE OBSERVATORY Department: STATISTICAL INVESTIGATIONS AND ANALYZES DEPARTMENT Address: 3 AVENUE DU STADE DE FRANCE Postal code: 93200 City: LA PLAINE SAINT DENIS RECEIPT dated February 21, 2018

Purpose: SURVEY ON ADOLESCENT BEHAVIOR Reported treatment Reporting body MUST READ The delivery of this receipt certifies that you have sent the CNIL a formally complete declaration file. You can now carry out your processing of personal data. The CNIL may at any time verify, by mail, by means of an on-site or online inspection, that this processing complies with all the provisions of the law of January 6, 1978 amended in 2004.

In order to comply with the law, you are required to respect the obligations provided for throughout your processing and in particular: 1) The definition and respect of the purpose of the processing, 2) The relevance of the data processed, 3) The retention for a limited period of the data, 4) Security and confidentiality of data, 5) Respect for the rights of interested parties: information on their right of access, rectification and opposition. For more details on the obligations provided for by the "information technology and freedoms" law, consult the CNIL website: www.cnil.fr SIREN or SIRET number: 180036105 00039 NAF or APE code: 8411Z Tel. : 0141627716 Fax. : 014162770

Purpose: SURVEY ON ADOLESCENT BEHAVIOR from the HSBS Frnch surveys 2010-2011 and 2017-2018.

21-02-2018

Information note for parents

An international survey by the World Health Organization on the health and lifestyles of students aged 11, 13 and 15-17: Health behavior in school-aged children (HBSC)

Dear, Madam, Sir,

Since 1982, the World Health Organization has organized a survey of young people aged 11, 13 and 15 every four years in order to better understand their lifestyles and health.

This year, the survey will be carried out in the same way in 35 other countries.

Your child is one of 10,000 students from classes drawn at random to represent France.

This survey, which is strictly anonymous, focuses mainly on lifestyle, sleep, diet, health, schooling, leisure activities and psychosocial and psycho-affective aspects of the child's life. After completing their questionnaire in class, each student will seal it and give it to an authorized investigator who is not part of the educational team of the school.

As in previous years, the overall results of the survey will be published in a national and international report.

However, if you do not want your child to participate in this survey, please send the coupon below to the head of the establishment by return post after having duly completed it.

I, the undersigned),

Last name First Name :

Do not wish that my child,

Last name First Name :

student of (specify class):

Participates in the WHO survey on young people's lifestyles.

Date and signature :

Site : https://www.em-consulte.com/em/livre/470195/TP9_p_107.pdf
