# Supplementary material for: Two surveys separated by almost a decade reveal the inexorable decline in sleep time in teens: Results of the National survey of middle and high schools in adolescents on health and substances (EnCLASS 2018), and the evolution since 2010
Source: PLoS One. 2025 Mar 31;20(3):e0314815. doi: 10.1371/journal.pone.0314815 (PMC11957357; doi:10.1371/journal.pone.0314815)
Supplement: Attachment — Submitted filename: PONE D24 21580 Leger D. rebutal letter.docx [file pone.0314815.s002.docx]

PONE-D-24-21580: Inexorable decline of sleep in teens: Answer to reviewers

1. Please ensure that your manuscript meets PLOS ONE's style requirements, including those for file naming. The PLOS ONE style templates can be found at
   <https://journals.plos.org/plosone/s/file?id=wjVg/PLOSOne_formatting_sample_main_body.pdf> and 
   <https://journals.plos.org/plosone/s/file?id=ba62/PLOSOne_formatting_sample_title_authors_affiliations.pdf>

We carefully checked, and followed the instructions.

2. We note that the grant information you provided in the ‘Funding Information’ and ‘Financial Disclosure’ sections do not match.

When you resubmit, please ensure that you provide the correct grant numbers for the awards you received for your study in the ‘Funding Information’ section.

Done

3. Thank you for stating the following in the Acknowledgments Section of your manuscript: 
"The authors are grateful to the French Ministry of Education (direction générale de l’enseignement 
scolaire, DGESCO ; direction des études de la performance et de la prospective, DEPP), Santé Publique 
France and OFDT. The authors are indebted to all of the participating children and adolescents and 
their parents, and to the teachers who accommodated the survey during their classes."

We note that you have provided funding information that is not currently declared in your Funding Statement. However, funding information should not appear in the Acknowledgments section or other areas of your manuscript. We will only publish funding information present in the Funding Statement section of the online submission form. 
Please remove any funding-related text from the manuscript and let us know how you would like to update your Funding Statement. Currently, your Funding Statement reads as follows: 
"The authors are grateful to the French Ministry of Education (direction générale de l’enseignement scolaire, DGESCO ; direction des études de la performance et de la prospective, DEPP), Santé Publique France and OFDT."

OK agree

Please include your amended statements within your cover letter; we will change the online submission form on your behalf.

4. We note that your Data Availability Statement is currently as follows: All relevant data are within the manuscript and its Supporting Information files.

Please confirm at this time whether or not your submission contains all raw data required to replicate the results of your study. Authors must share the “minimal data set” for their submission. PLOS defines the minimal data set to consist of the data required to replicate all study findings reported in the article, as well as related metadata and methods (<https://journals.plos.org/plosone/s/data-availability#loc-minimal-data-set-definition>).

For example, authors should submit the following data:

- The values behind the means, standard deviations and other measures reported;
- The values used to build graphs;
- The points extracted from images for analysis.

Authors do not need to submit their entire data set if only a portion of the data was used in the reported study.

All relevant data are within the manuscript.

If your submission does not contain these data, please either upload them as Supporting Information files or deposit them to a stable, public repository and provide us with the relevant URLs, DOIs, or accession numbers. For a list of recommended repositories, please see <https://journals.plos.org/plosone/s/recommended-repositories>.

If there are ethical or legal restrictions on sharing a de-identified data set, please explain them in detail (e.g., data contain potentially sensitive information, data are owned by a third-party organization, etc.) and who has imposed them (e.g., an ethics committee). Please also provide contact information for a data access committee, ethics committee, or other institutional body to which data requests may be sent. If data are owned by a third party, please indicate how others may request data access.

There is no restriction.

5. Please include captions for your Supporting Information files at the end of your manuscript, and update any in-text citations to match accordingly. Please see our Supporting Information guidelines for more information: <http://journals.plos.org/plosone/s/supporting-information>.

Table 2 supplementary is positioned at the end of the manuscript.

6. Please review your reference list to ensure that it is complete and correct. If you have cited papers that have been retracted, please include the rationale for doing so in the manuscript text, or remove these references and replace them with relevant current references. Any changes to the reference list should be mentioned in the rebuttal letter that accompanies your revised manuscript. If you need to cite a retracted article, indicate the article’s retracted status in the References list and also include a citation and full reference for the retraction notice.

The reference list is revised.

[Note: HTML markup is below. Please do not edit.]

Reviewers' comments:

Reviewers' comments:

Reviewer's Responses to Questions

**Comments to the Author**

1. Is the manuscript technically sound, and do the data support the conclusions?

The manuscript must describe a technically sound piece of scientific research with data that supports the conclusions. Experiments must have been conducted rigorously, with appropriate controls, replication, and sample sizes. The conclusions must be drawn appropriately based on the data presented.

Reviewer #1: Yes

Reviewer #2: Yes

2. Has the statistical analysis been performed appropriately and rigorously?

Reviewer #1: Yes

Reviewer #2: Yes

3. Have the authors made all data underlying the findings in their manuscript fully available?

The [PLOS Data policy](http://c05y1x9s.r.us-east-2.awstrack.me/L0/http:%2F%2Fwww.plosone.org%2Fstatic%2Fpolicies.action%23sharing/1/010f01917630e831-5e7cacbd-9aad-4ac2-9f35-c110753fa61e-000000/bJ5Ni_cSu8EvglzScvhDXbGJseQ=173) requires authors to make all data underlying the findings described in their manuscript fully available without restriction, with rare exception (please refer to the Data Availability Statement in the manuscript PDF file). The data should be provided as part of the manuscript or its supporting information, or deposited to a public repository. For example, in addition to summary statistics, the data points behind means, medians and variance measures should be available. If there are restrictions on publicly sharing data—e.g. participant privacy or use of data from a third party—those must be specified.

Reviewer #1: Yes

Reviewer #2: Yes

4. Is the manuscript presented in an intelligible fashion and written in standard English?

PLOS ONE does not copyedit accepted manuscripts, so the language in submitted articles must be clear, correct, and unambiguous. Any typographical or grammatical errors should be corrected at revision, so please note any specific errors here.

Reviewer #1: Yes

Reviewer #2: Yes

**5. Review Comments to the Author**

Please use the space provided to explain your answers to the questions above. You may also include additional comments for the author, including concerns about dual publication, research ethics, or publication ethics. (Please upload your review as an attachment if it exceeds 20,000 characters)

**Reviewer #1**: The authors present results of an interesting epidemiological study on the temporal dynamics of sleep behavior in French schoolchildren. The methodology is good and the results are solid. My only concern is about the English in this manuscript.

For example, the following sentence sound strange for me:

"From October 1 2010 to June 30 2011 for the first time, we documented in this Journal the sleep of French adolescents using the Health Behaviour in School-aged Children (HBSC/WHO) survey [5-6] and the 2011 (April 1 to June 30) European School Survey Project on Alcohol and Other Drugs (ESPAD) survey".

What do you mean by saying "we documented in this Journal”?

R: We wanted to say, “we published in Plos-One”. We revised it:

“Based on data collected from October 1, 2010 to June 30, 2011, we document here for the first time, we documented in this Journal the sleep of French adolescents using the Health Behaviour in School-aged Children (HBSC/WHO) survey [5-6] and the 2011 (April 1 to June 30) European School Survey Project on Alcohol and Other Drugs (ESPAD) survey [6]”

I recommend having the manuscript revised linguistically by a native speaker.

The manuscript has been revised by a native speaker (see all corrections on the redline manuscript, tables and highlights.

**Reviewer #2:** The study compares data from a large representative survey conducted 8 years apart on juvenile sleep behavior and sleep parameters in France. The data is highly interesting and relevant to current study questions. I just have a few comments

Abstract
The term "total sleep time" is typically utilized in the context of polysomnography, where it is defined in a specific manner. This term is employed throughout the manuscript, indicating the presence of objective sleep data that were not collected in this study. I suggest the term sleep duration instead.

In the method part, we concisely define total sleep time TST based on meticulous sleep logs as the difference between the time at which the participant went to bed and the time of day that they woke up, discounting the time needed to fall asleep. This definition is indeed close from the one obtained with objective polysomnography. We believe that sleep duration is too vague and may be confused with the subjective feeling of participants. This is why we propose to keep TST.

The terms sleep debt and too short sleep should already be defined in the methods section.

Thank-you, we introduced the definitions in the abstract method part: Sleep debt as was defined as a difference between the total sleep time (TST) during schooldays (TSTS) non-schooldays (weekends or vacations; TSTN) of over 2 hours. Too short sleep was assesses when TSTS was < 7 hours.

Results: The term “lost” is too interpretative. “Increase of 36.8. points” is not understandable because it was not explained beforehand.

Agree, we replace it by “the TSTS of middle schoolers decreases by an average of 20 minutes of sleep per night on weekdays, dropping from 8 h 35 min to 8 h 14 min.”

Conclusion: A forward-looking conclusion would be desirable here.

Thank-you, we replaced the conclusion by : “Faced with this trend, teachers and parents need to take preventive action to avoid an inexorable decline in teenagers' sleep.”

Introduction
“These activities are a major reason.....” Please provide one or more literature sources for this statement.

Thank-you, we added these two references:

Owens J; Adolescent Sleep Working Group; Committee on Adolescence. Insufficient sleep in adolescents and young adults: an update on causes and consequences. Pediatrics. 2014 Sep;134(3):e921-32.

Dresp-Langley B, Hutt A. Digital Addiction and Sleep. Int J Environ Res Public Health. 2022 Jun 5;19(11):6910. doi: 10.3390/ijerph19116910.
“The statistical department of the French Ministry...the international requirements” Please provide a source here.

Done.
Statistical analyses
“...using National data...” Please indicate reference.

Done.

Results
“Impressive rates.....” The word is a little tendentious

Agree replaced by “high”.

Discussion
“To our knowledge...............” This sentence is somewhat confusing, as it is followed by a list of various studies on precisely this topic

Agree, the sentence was replaced by “Our study was specifically designed to assess the epidemiology of TST in a large number of children and adolescents”

Regarding the limitations
One limitation is certainly that the subjective quality of sleep was not assessed. It would be interesting to know whether the students also perceived their sleep as too short.

Agree we have added this limitation: “Third, we did not interview children on how they perceived their sleep was too short or not.”

Additionally, the last sentence of the conclusion could be more specific to the results of this study, as it is currently formulated in a way that applies to all possible results in different areas.

Thank you, we replace the last sentence by this most focused one: “Given these results, we hope that educators, parents and health authorities will be able to make sleep a priority in the health education given to children, by making them understand how limiting their sleep can have consequences for their quality of life and health.”
